# Molecular Characterization of Methicillin Resistant *Staphylococcus aureus* Strains Isolated from Intensive Care Units in Iran: ST22-SCC*mec* IV/t790 Emerges as the Major Clone

**DOI:** 10.1371/journal.pone.0155529

**Published:** 2016-05-12

**Authors:** Mehdi Goudarzi, Hossein Goudarzi, Agnes Marie Sá Figueiredo, Edet E. Udo, Maryam Fazeli, Mohammad Asadzadeh, Sima Sadat Seyedjavadi

**Affiliations:** 1 Department of Microbiology, School of Medicine, Shahid Beheshti University of Medical Sciences, Tehran, Iran; 2 Departamento de Microbiologia Medica, Instituto de Microbiologia Paulo de Goes, Centro de Ciencias da Saude, Universidade Federal do Rio de Janeiro, Rio de Janeiro, Brazil; 3 Department of Microbiology, Faculty of Medicine, Kuwait University, Safat, Kuwait; 4 WHO Collaborating Center for Reference and Research on Rabies, Pasteur Institute of Iran, Tehran, Iran; 5 Department of Medical Mycology, Pasteur institute of Iran, Tehran, Iran; Chang Gung Memorial Hospital, TAIWAN

## Abstract

**Introduction:**

The emergence of methicillin-resistant *Staphylococcus aureus* (MRSA) in different patient populations is a major public health concern. This study determined the prevalence and distribution of circulating molecular types of MRSA in hospitalized patients in ICU of hospitals in Tehran.

**Materials and Methods:**

A total of 70 MRSA isolates were collected from patients in eight hospitals. Antimicrobial resistance patterns were determined using the disk diffusion method. The presence of toxin encoding genes and the vancomycin resistance gene were determined by PCR. The MRSA isolates were further analyzed using multi-locus sequence, *spa*, SCC*mec*, and *agr* typing.

**Results:**

The MRSA prevalence was 93.3%. Antimicrobial susceptibility testing revealed a high resistance rate (97.1%) to ampicillin and penicillin. The rate of resistance to the majority of antibiotics tested was 30% to 71.4%. Two isolates belonging to the ST22-SCC*mec* IV/t790 clone (MIC ≥ 8 μg/ml) had intermediate resistance to vancomycin. The majority of MRSA isolates (24.3%) were associated with the ST22-SCC*mec* IV/t790 clone; the other MRSA clones were ST859-SCC*mec* IV/t969 (18.6%), ST239-SCC*mec* III/t037 (17.1%), and ST291-SCC*mec* IV/t030 (8.6%).

**Conclusions:**

The circulating MRSA strains in Iranian hospitals were genetically diverse with a relatively high prevalence of the ST22-SCC*mec* IV/t790 clone. These findings support the need for future surveillance studies on MRSA to better elucidate the distribution of existing MRSA clones and detect emergence of new MRSA clones.

## Introduction

*Staphylococcus aureus* is a versatile and dangerous human pathogen equipped with many virulence factors and is the major cause of important infections in community and hospital settings [1]. *S*. *aureus* is responsible for infections ranging from folliculitis, food poisoning, osteomyelitis, endocarditis, septic arthritis, pneumonia, and skin and deep tissue infections to life-threatening invasive diseases [2]. It is well-established that *S*. *aureus* infections in hospitals and health care institutions show an increasing prevalence of methicillin-resistant *S*. *aureus* (MRSA) isolates. They are usually resistant to macrolides, lincosides, aminoglycoside, and approximately all currently available beta-lactam antimicrobial agents [3–5].

MRSA is a major cause of morbidity and mortality both in healthcare settings and in healthy individuals in the last two decades. The global emergence and spread of MRSA harboring multi-resistance genes limits the effectiveness of therapeutic options for staphylococcal infections and worsens their clinical outcomes [6]. It is evident that methicillin resistance primarily results from the expression of low-affinity penicillin-binding protein (PBP) genes namely *mecA*. The *mec* genes are located on a region of the staphylococcal cassette chromosome (SCC) and 11 types of SCC*mec* have been characterized thus far [7].

The distinction between hospital-associated MRSA (HA-MRSA) and community-associated MRSA (CA-MRSA) is becoming blurred as transmission of *S*. *aureus* from the community to hospitals and vice versa easily occurs. There is evidence that USA300, a dominant CA-MRSA clone in North America, has invaded healthcare facilities and established itself as a nosocomial pathogen. HA-MRSA is generally distinguished from CA-MRSA based on epidemiological, phenotypic, and genotypic characteristics [8, 9]. Molecular typing is an established tool for studying the relatedness of MRSA strains, their clonal distribution, genetic diversity, and transmission modes. Various techniques have been developed to type MRSA isolates, including pulsed-field gel electrophoresis (PFGE), multilocus sequence typing (MLST), staphylococcal cassette chromosome mecA (SCC*mec*), coa typing, *spa*-typing by sequencing the highly polymorphic staphylococcus protein A (*spa*) gene, the *mec*-associated hypervariable region (dru), and the accessory gene regulator (*agr*) [1, 3, 10].

Although PFGE is usually considered when investigating MRSA outbreaks, sequencing techniques, especially *spa* typing has emerged as an effective and rapid method for typing MRSA to moderate discrimination and exhibits high throughput and good inter-laboratory reproducibility. *Spa* typing is based on the number of tandem repeats and the sequence variation in region X of the protein A gene [11]. It has been documented that a combination of genetic background and SCC*mec* typing should be used to describe MRSA clones [12].

The prevalence of MRSA infections among hospitalized patients in ICUs has increased steadily in Iran. Unfortunately, information about the distribution of MRSA *spa* types and MLST in Iran is sparse. The present study determined the MRSA prevalence among hospitalized patients in ICUs and identified the molecular types of MRSA.

## Material and Methods

### Study design and population

This cross-sectional study was conducted from November 2014 to July 2015 in eight hospitals: two in northern Tehran (A1 and A2), two in central Tehran (B1 and B2), two in southern Tehran (C1 and C2), one in eastern Tehran (D1), and one in western Tehran (D2). All participants were ICU patients with *S*. *aureus* infections. The demographic characteristics of age, gender, department and period of hospitalization, antibiotic usage, and underlying conditions were recorded. Written informed consent was obtained from all patients and the study protocol was approved by the Ethics Committee of Shahid Beheshti University of Medical Sciences (No 6981).

A case of healthcare-associated MRSA was considered as being hospital onset (HO) if a positive culture of MRSA was obtained on or after 96 hours of admission to a hospital. It was considered as community onset (CO) if the culture was obtained before the fourth calendar day of hospitalization and had one of more of the following features: (1) a history of hospitalization, surgery, dialysis, or residence in a long-term care facility within 12 months preceding the culture date, or (2) the presence of a central vascular catheter (CVC) within the 2 days prior to MRSA culture.

A case of invasive MRSA infection was defined by the isolation of MRSA from normally sterile body sites including the blood, cerebrospinal fluid, pleural fluid, pericardial fluid, peritoneal fluid, joint/synovial fluid, bone, internal body sites (lymph node, brain, heart, liver, spleen, vitreous fluid, kidney, pancreas, or ovary), or other normally sterile sites. MRSA obtained from any clinical site was chosen for determination of antibiotic resistance, virulence factors, and molecular analysis. Duplicate isolates from the same patients and MRSA isolates that were not clinical isolates were excluded from the present survey.

### Bacterial strains

All samples obtained from hospitalized ICU patients were transported to the laboratory within four hours of collection and processed immediately. Isolates were identified to the species level as *S*. *aureus* using standard microbiological procedures such as gram staining, growth patterns on mannitol salt agar, catalase testing, rabbit plasma coagulase testing, and DNase testing. To definitively identify positive *S*. *aureus* isolates, they were subjected to polymerase chain reaction (PCR) for *femA* and *nucA* genes. The MRSA isolates were screened using a cefoxitin disc (30 μg) on Mueller-Hinton agar plates supplemented with 4% NaCl and were confirmed by the amplification of the *mecA* gene by PCR. The MRSA isolates were stored in Tryptic soy broth (TSB; Merck; Germany) containing 20% glycerol at -70°C for further molecular investigation.

### Antimicrobial susceptibility testing of MRSA isolates

Susceptibility to vancomycin (VA; 30 μg), ampicillin (AP; 10 μg), kanamycin (K; 30 μg), ciprofloxacin (CIP; 5μg), clindamycin (CD; 2 μg), linezolid (LZD; 30 μg), penicillin (PG; 10 μg), teicoplanin (TEC; 30 μg), amikacin (AK; 30 μg), tobramycin (TN; 10 μg), gentamicin (GM; 10 μg), trimethoprim-sulfamethoxazole (TS; 2.5 μg) and ceftriaxon (CRO; 30 μg) was determined for all MRSA isolates by Kirby-Bauer disk diffusion in accordance with Clinical and Laboratory Standards Institute (CLSI) recommendations [13]. The minimum inhibitory concentration (MIC) for vancomycin was determined with E-test strips (AB Biodisk; Sweden) according to manufacturer instructions. Multidrug resistance (MDR) was defined as resistance of MRSA to three or more unique antimicrobial drug classes in addition to beta-lactams. All antibiotic disks used in this research were supplied by Mast (UK). The standard reference strain *S*. *aureus* ATCC25923 was used as a quality control strain in every test run.

### Extraction of genomic DNA

Genomic DNA of MRSA strains were extracted using the commercial kit InstaGene Matrix (BioRad; USA) with the addition of lysostaphin (Sigma–Aldrich; USA) to a final concentration of 15 μg/ml. After DNA extraction, the concentration of DNA was assessed using a spectrophotometer.

### Detection of toxin encoding genes, *vanA* gene

All 70 MRSA isolates were tested for the presence of lukS-PV-lukF-PV (*pvl* genes), toxic shock syndrome toxin (*tsst*) gene, and *vanA* gene with the degenerate primers listed in Table 1.

**Table 1:** Oligonucleotide primers used in this study.

Target	primer	Primer sequence (5´→3´)	Product size (bp)	Reference
*femA*	F	CTTACTTACTGCTGTACCTG	648	[14]
	R	ATCTCGCTTGTTGTGTGC		
*nucA*	F	GCGATTGATGGTGATACGGTT	270	[15]
	R	AGCCAAGCCTTGACGAACTAAAGC		
*mecA*	F	AGAAGATGGTATGTGGAAGTTAG	583	[16]
	R	ATGTATGTGCGATTGTATTGC		
*luk-PV*	F	TTCACTATTTGTAAAAGTGTCAGACCCACT	180	[17]
	R	TACTAATGAATTTTTTTATCGTAAGCCCTT		
*tsst-1*	F	TTATCGTAAGCCCTTTGTTG	398	[16]
	R	TAAAGGTAGTTCTATTGGAGTAGG		
*VanA*	F	GGCAAGTCAGGTGAAGATG	713	[16]
	R	ATCAAGCGGTCAATCAGTTC		
*agr*	Pan F	ATGCACATGGTGCACATGC	-	[18]
	R1	GTCACAAGTACTATAAGCTGCGAT	441	[18]
	R2	TATTACTAATTGAAAAGTGGCCATAGC	575	[18]
	R3	GTAATGTAATAGCTTGTATAATAATACCCAG	323	[18]
	R4	CGATAATGCCGTAATACCCG	659	[18]
SCC*mec*	Fβ	ATTGCCTTGATAATAGCCYTCT	937	[7]
	R α3	TAAAGGCATCAATGCACAAACACT		
	F ccrC	CGTCTATTACAAGATGTTAAGGATAAT	518	[19]
	R ccrC	CCTTTATAGACTGGATTATTCAAAATAT		
	F 1272	GCCACTCATAACATATGGAA	415	[7, 20]
	R 1272	CATCCGAGTGAAACCCAAA		
	F 5R*mec*A	TATACCAAACCCGACAACTAC	359	[19, 20]
	R 5R431	CGGCTACAGTGATAACATCC		
	F	ATCATTAGGTAAAATGTCTGGACATGATCCA	433	[17]
	R	GCATCAAGTGTATTGGATAGCAAAAGC		

### Determination of accessory gene regulator (*agr*) type

The detection of *agr* types was carried out for all 70 MRSA isolates using the multiplex PCR and primer set comprising a common forward primer (Pan) and reverse primers (*agr*1, *agr*2, *agr*3, and *agr*4) specific to each *agr* group. These primers were designed to amplify a 441-bp fragment of the *agr* group I strains, a 575-bp fragment of the *agr* group II strains, a 323-bp fragment of the *agr* group III strains, and a 659-bp fragment of the *agr* group IV strains. The primer sequences are listed in Table 1.

### SCC*mec* typing

SCC*mec* typing was performed for all MRSA isolates by multiplex PCR as described previously. Primer sequences are showed in Table 1. SCC*mec* types were identified by comparing the banding patterns of MRSA to ATCC 10442 (SCC*mec* type I), N315 (SCC*mec* type II), 85/2082 (SCC*mec* type III), MW2 (SCC*mec* type IVa), WIS (SCC*mec* type V) as reference strains.

### Typing of the *S*. *aureus* protein A locus

*Spa* typing was performed as described by Harmsen et al. [11] for all MRSA isolates. Amplified fragments were subjected to DNA sequencing of both amplicon strands by Macrogen (South Korea). The isolates were assigned to the specific *spa* types according to the guidelines described by a Ridom SpaServer database (http://www.spaserver.ridom.de).

### Multi-locus sequence typing (MLST)

MLST with standard primers introduced by the MLST database was performed on all isolates based on seven housekeeping genes (*arc*C, *aro*E, *glp*F, *gm*K, *pta*, *tpi*A and *yqi*L) as described by Enright et al. Isolates were assigned a sequence type (ST) according to the MLST website (http://www.mlst.net/).

### Nucleotide sequencing

Amplified PCR products were purified with QIAquick Gel Extraction Kit and then were sequenced with an ABI Prism 377 automated sequencer (Applied Biosystems; Perkin-Elmer; USA) in both directions. The sequences were used for both confirmation and sequence-based typing methods (MLST and *spa* typing).

## Results

A total of 75 *S*. *aureus* isolates were obtained from 350 infected ICU patients during the 8-month study period. Of these patients, 45 (64.3%) were female and 25 (35.7%) were male. The median age of the patients was 46.8 years (11 months to 69 years). Of the 75 *S*. *aureus* isolates, 70 (93.3%) were MRSA. The frequency of MRSA isolated from the hospitals in Tehran was 20 MRSA isolates (28.6%) from hospitals C1 and C2, 15 MRSA isolates (21.4%) from hospitals A1 and A2, 15 MRSA isolates (21.4%) from hospitals B1 and B2, 10 isolates (14.3%) from hospital D1, and 10 isolates (14.3%) from hospital D2. The MRSA isolates were recovered from wounds (n = 21, 30%), as skin abscesses (14.3%), surgical wounds (7.1%), decubitus wounds (4.3%), and traumatic wounds (4.3%), blood (n = 15, 21.4%), sputum (n = 14, 20%), the ear (n = 8, 11.4%), exudate/pus (n = 4, 5.7%), a catheter (n = 3, 4.3%), body fluids (bronchoalveolar lavage and cerebrospinal fluid; n = 3, 4.3%) and urine (n = 2, 2.9%).

Among the MRSA isolates, 34.3% were classified as CO and 65.7% as HO. The rate of invasive MRSA was 25.7% and was higher among HO cases than CO cases. The occurrence of infections with MRSA was highest (64.3%) in the 25–45 year age group and lowest (4.3%) in the ≤15 years of age group. Subsequently, all MRSA isolates were subjected to antimicrobial susceptibility testing. It was found that 97.1% of isolates were resistant to ampicillin or penicillin. The rates of resistance to the majority of antibiotics tested varied from 30% to 71.4%. Intermediate-resistance to vancomycin was observed in two isolates (MIC ≥ 8 μg/ml), both belonging to agr group II. Linzolid, vancomycin, teicoplanin, and ceftriaxon showed good activity against MRSA isolates. Of the 70 MRSA isolates, 40 (57.1%) were MDR. *In vitro* susceptibility of the MRSA isolates to the 13 tested antibiotics is shown in Table 2. CO–MRSA isolates were more susceptible to multiple antibacterial classes than HO isolates. The predominant MDR profile among the isolates included a resistance profile to six antibiotics (31.4%), seven antibiotics (24.3%), five antibiotics (18.6%), eight antibiotics (14.3%), and four antibiotics (11.4%).

**Table 2:** Antimicrobial susceptibility profile of 70 health care-associated MRSA isolates to 13 antimicrobial agents.

Type of antibiotic	No (%) resistance	No (%) resistance	Total resistance No (%)
	Community-onset	Hospital onset	
penicillin	22(31.4)	46(65.7)	68(97.1)
ampicilin	13(18.5)	55(78.6)	68(97.1)
vancomycin	0(0)	0(0)	0(0)
teicoplanin	0(0)	0(0)	0(0)
ceftriaxon	1(1.5)	5(7.1)	6(8.6)
gentamicin	10(14.3)	32(45.7)	42(60)
kanamycin	12(17.1)	28(40)	40(57.1)
amikacin	13(18.6)	32(45.7)	45(64.3)
tobramycin	8(11.4)	32(45.7)	40(57.1)
linzolid	0(0)	0(0)	0(0)
clindamycin	20(28.6)	22(31.4)	42(60)
ciprofloxacin	3(4.3)	47(67.1)	50(71.4)
trimetoprim-sulfamethoxazole	13(18.6)	8(11.4)	21(30)

Six different profiles (ST22 in 24 strains, ST859 in 16 strains, ST239 in 12 strains, ST291 in six strains, ST30 in four strains, and ST6 in eight strains) were identified by MLST among the isolates. All MRSA isolates were *spa* typed and the results discriminated nine *spa* types (t790 in 18 strains; t969 in 16 strains; t030 in six strains; t037 in 14 strains, t034 in eight strains; t7580 in three strains; t1425 in three strains; and t230 in two strains). The majority of sequence types and *spa* types were disproportionately distributed between CO and HO isolates (Table 3).

**Table 3:** Epidemiological and molecular characteristics of 70 MRSA isolates in present study.

Characteristic	Community-onset n(%)	Hospital-onset n(%)	Total n(%)
Agr			
I	20(28.6)	25(35.7)	45(64.3)
II	1(1.4)	11(15.7)	12(17.1)
III	6(8.6)	0(0)	6(8.6)
IV	1(1.4)	2(2.9)	3(4.3)
SCCmec			
IV	24(34.3)	19(27.1)	67(61.4)
III	0(0)	21(30)	21(30)
II	0(0)	6(8.6)	6(8.6)
Sequence types			
22	8(11.4)	16(22.9)	24(34.3)
859	8(11.4)	8(11.4)	16(22.8)
239	0(0)	12(17.1)	12(17.1)
291	3(4.3)	3(4.3)	6(8.6)
30	3(4.3)	1(1.4)	4(5.7)
6	2(2.9)	6(8.6)	8(11.5)
spa types			
t790	8(11.4)	10(14.3)	18(25.7)
t969	8(11.4)	8(11.4)	16(22.8)
t030	3(4.3)	3(4.3)	6(8.6)
t037	0(0)	14(20)	14(20)
t034	2(2.9)	2(2.9)	4(5.8)
t7580	0(0)	3(4.3)	3(4.3)
t1425	3(4.3)	0(0)	3(4.3)
t230	0(0)	2(2.9)	2(2.9)
PVL	15(21.4)	0(0)	15(21.4)
TSST	20(28.6)	16(22.9)	36(51.5)

The majority of MRSA strains carried SCC*mec* IV (43 strains, 61.4%), with *spa* types t790 (n = 16, 37.3%), t969 (n = 13, 30.2%), t030 (n = 6, 13.9%), t034 (n = 4, 9.3%), t1425 (n = 3, 7%) and t7580 (n = 1, 2.3%). The second most frequent type was SCC*mec* III (21 strains, 30%) with *spa* types t037 (n = 14, 66.7%), t969 (n = 3, 14.3%), t7580 (n = 2, 9.5%) and t230 (n = 2, 9.5%). SCC*mec* II was found in six strains (8.6%) with *spa* types t034 (n = 4, 66.7%) and t790 (n = 2, 33.3%). The existence of SCC*mec* type I was not confirmed in any MRSA strain. The *agr* typing identified four types in 70 MRSA isolates: *agr* group I (45, 64.3%), II (12, 17.1%), III (6; 8.6%) and type IV (3; 4.3%) alleles. Note that four isolates (5.7%) (two ST291, one ST22, and one ST239) could not be assigned to a known *agr* type.

Fifteen isolates (21.4%) were positive for *pvl*-encoding genes. All 15 isolates carrying the *pvl* genes belonged to the ST22-SCC*mec* IV/t790 clone. The PCR results of MRSA isolates revealed that 36 (51.4%) carried the *tsst* gene. The *tsst* gene was identified in six STs (ST22, ST291, ST239, ST6, ST859, and ST30). Table 4 lists the characteristics of the MRSA molecular types investigated.

**Table 4:** Distribution of MRSA molecular types by invasive and non-invasive infection.

Molecular types	Invasive MRSA		Non-invasive MRSA		Total n(%)
	Community-onset n(%)	Hospital-onset n(%)	Community-onset n(%)	Hospital-onset n(%)	
ST22-SCC*mec* IV/t790	4(5.7)	1(1.4)	4(5.7)	7(10)	16(22.8)
ST859-SCC*mec* IV/t969	2(2.9)	1(1.4)	6(8.6)	4(5.7)	13(18.5)
ST239-SCC*mec* III/t037	0(0)	1(1.4)	0(0)	11(15.7)	12(17.1)
ST291-SCC*mec* IV/t030	0(0)	1(1.4)	3(4.3)	2(2.9)	6(8.6)
ST6-SCC*mec* IV/t034	2(2.9)	1(1.4)	0(0)	1(1.4)	4(5.7)
ST6-SCC*mec* II/t034	0(0)	1(1.4)	0(0)	3(4.3)	4(5.7)
ST859-SCC*mec* III/t969	0(0)	1(1.4)	0(0)	2(2.9)	3(4.3)
ST30-SCC*mec* IV/t1425	0(0)	0(0)	3(4.3)	0(0)	3(4.3)
ST22-SCC*mec* III/t037	0(0)	0(0)	0(0)	2(2.9)	2(2.9)
ST22-SCC*mec* III/t7580	0(0)	0(0)	0(0)	2(2.9)	2(2.9)
ST22-SCC*mec* III/t230	0(0)	0(0)	0(0)	2(2.9)	2(2.9)
ST22-SCC*mec* II/t790	0(0)	2(2.9)	0(0)	0(0)	2(2.9)
ST30-SCC*mec* IV/t7580	0(0)	1(1.4)	0(0)	0(0)	1(1.4)

### ST22-SCC*mec* IV/t790

Approximately two-thirds of patients infected with the ST22-SCC*mec* IV/t790 clone were in the 25–45 year age group. This strain was isolated from hospitals A1, A2, B1, and B2. All but one harbored the PVL-encoding gene for toxins and were positive (with the exception of one isolate) for the *tsst* gene. The majority of this strain belonged to *agr* group I. These strains were obtained from wounds (43.8%), blood (31.2%), sputum (18.8%), and ears (6.2%). All strains of this clone were remarkably MDR. The resistance profile of the ST22-SCC*mec* IV/t790 strains included resistance to eight (35.3%), seven (17.6%), 6 (23.5%), five (5.9%), and four (17.7%) antibiotics. All of these isolates were simultaneously resistant to ampicillin, penicillin, and ciprofloxacin. Interestingly, two isolates showing intermediate-resistance to vancomycin with MIC ≥ 8 μg/ml were isolated from hospitals A1 and A2.

### ST859-SCC*mec* IV/t969

ST859-SCC*mec* IV/t969 was the second most-common MRSA clone identified in this study. All but two patients fell into the 25–45 year age group. This strain was isolated from hospitals C1, C2, and D1. The frequency of *tsst* in this clone was low (15.4%) and the *pvl*-encoding gene was not confirmed in any ST859-SCC*mec* IV/t969 strains.

## Discussion

Nosocomial *S*. *aureus* infections caused by MRSA strains have emerged globally as an important clinical pathogen, including in Iran. HA-MRSA results in severe morbidity and mortality in ICUs worldwide [2, 6]. ICUs are high-risk areas for MRSA infections, which requires further investigation. Given the limited knowledge about the prevalence and distribution of MRSA genotypes in this part of Asia, an attempt was made to characterize MRSA isolated from ICUs. In line with other studies [21] showing an increased frequency of MRSA in ICUs, MRSA screening revealed that the prevalence of MRSA isolates was 93.3%. The high frequency of HA-MRSA isolates in the present study were comparable to the results of studies in Bolivia [22], Serbia [23], Nigeria [24], Taiwan [10], Egypt [25], and Croatia [12], The reasons for the discrepancy in MRSA prevalence could be related to the origin of the isolates and the characteristics of the participants and hospital wards.

It has been reported that HA-MRSA exhibits different genetic characteristics in different geographic regions. Several clones seem to have emerged in Europe. For example, the ST8, ST247, ST239 and ST228 clones are predominant in Italy [9]. Multi-resistant HA-MRSA is characterized by the ST111-MRSA-I clone in Croatia [12], ST8-MRSA IV in France [26], and ST228 I and ST5 II in Hungary [2]. There is also a diversity of HA-MRSA clones in Asia. Asian countries exhibit the most predominant clones: ST6-IV/t304 clone in Oman [27], ST239-MRSA III in Saudi Arabia [28], ST239-MRSA IV in Iran [29], ST239-MRSA II in China, India and Indonesia, ST5-MRSA II in Korea and Japan [30], and ST30-MRSA IV in Qatar [31].

It has been established that SCC*mec* types I, II, III are related to HA-MRSA while the SCC*mec* types IV and V are prominent types of CA-MRSA [20]. In this survey, the SCC*mec* type IV was the most prominent type (61.4%) among MRSA isolates. The high frequency of SCC*mec* IV in the current study supports the observation that SCC*mec* IV is probably more mobile than other SCC*mec* types. This speculation is further strengthened because most health care-associated MRSA infections in Tehran are caused by casual transmission of MRSA strains that originated in communities.

Different typing methods showed the the major clones circulating in Tehran hospitals belonged to the six genetic background types (ST22, ST859, ST291, ST239, ST6, and ST30). HA-MRSA isolates belonging to the ST22-SCC*mec* IV/t790 clone were detected in 22.9% of the isolates as the dominant clone. ST22 is a global HA-MRSA pandemic clone that emerged in the UK in 1991 and is known as UK-EMRSA-15, Canadian MRSA-8, Barnim Epidemic Strain, Irish AR06, and Spanish PFGE type E13 [32]. Several studies have reported variable virulence markers in ST22 strains [32]. The high prevalence of ST22 among the clinical strains in Iran could result from MRSA transfer from the community to hospitals.

CA-MRSA strains typically share a type IV or V SCC*mec* and the PVL locus of different clones [23]. All but one ST22-SCC*mec* IV/t790 strain harbored the PVL-encoding gene. Additionally, all but one isolate that share the ST22-SCC*mec* IV/t790 strains were positive for the *tsst* gene. This finding is in agreement with a study in Abu Dhabi in which of 63 MRSA isolates, four were positive for tsst and another four were PVL positive [33]. *S*. *aureus* ST22 with PVL has been detected in Germany [34], England [6], Ireland [35], Saudi Arabia [28], Kuwait [36], and Qatar [31]. A study in England from 2005 to 2008 found that 76 MDR-MRSA isolates were PVL positive. Ellington et al. [6] showed that the PVL-MRSA strains belonged to ST772, ST5, ST8, ST22, ST59 and ST80 and multiple antibiotic resistance was identified among the STs. In India, Nadig et al. [37] demonstrated that All ST22 strains belonged to SCC*mec* IV and were positive for *pvl* and *tsst* encoding genes. The majority of EMRSA-15 isolates were also *spa* type t852 and t005.

Studies have shown that ST clones of MRSA often display different antibiotic resistance patterns [6, 27]. In the current study, isolates with ST22-SCC*mec* IV/t790 were significantly resistant to many antibiotic groups, particularly to penicillin, ampicilin, amikacin, gentamicin, and ciprofloxacin. This finding is in accordance with results of a study in England by Ellington et al. [6] that found PVL-MRSA strains belonging to ST types 772, 5, 8, 22, 59 and 80 that were consistently highly-resistant and multiple antibiotic-resistant. A study to determine the prevalence and distribution of MRSA genotypes circulating at a tertiary hospital in Oman reported that among 79 MRSA isolates, all ST22-IV strains were HA-MRSA, PVL positive, and multidrug-resistant [27]. In contrast to the present results, in a study in the city of Isfahan, Iran, 7 strains of ST22 methicillin-susceptible *S*. *aureus* (MSSA) were found as the causative agents of nosocomial infections and no ST22 MRSA was detected [38].

Vancomycin-resistant *S*. *aureus* (VRSA) and vancomycin-intermediate *S*. *aureus* (VISA) strains with a heterogeneous genetic background were reported in several studies [16]. In the United States, most VRSA strains isolated from clinical samples belonged to ST5. Surprisingly, in the present survey, two isolates with intermediate-resistance to vancomycin were related to the ST22-SCC*mec* IV/t790 clone. Recently, Azimian et al. [16] reported one VRSA isolate separated from the bronchial aspirate of a 26-year-old man with genetically characteristic SCC*mec* type III, *agr* I, *spa* type t037, and ST1283. Studies have shown that VRSA strains have certain differences in their ST, *spa*, and SCC*mec* types [39]. The existence of VISA and VRSA strains in Iran may be the result of selective pressure caused by inappropriate use of vancomycin in Iranian health care settings and hospitals for any *S*. *aureus* infection.

Several studies have shown that MRSA clones are steadily changing [2, 10]. During the 15-year period from 1994 to 2008, a total of 302 MRSA isolates were investigated in China and it was observed that the ST239-MRSA-III-*spa* t037 clone was replaced by the emerging ST239-MRSA-III-*spa* t030 clone [40]. A 10-year survey in Hungary to trace the temporal evolution of epidemic clones recovered a total of 238 MRSA isolates from 1994 to 1998 and 299 MRSA isolates from 2001 to 2004. From 1994 through 1998, ST239-MRSA-III was reported as the predominant MRSA clone; however, it had practically disappeared by 2003–2004 and was replaced with the Southern German clone (PFGE B, ST228-I) and the New York⁄Japan epidemic clone (PFGE A, ST5-II) [2].

The other two major clones, ST859-SCC*mec* IV/t969 and ST291-SCC*mec* IV/t030, have been most frequently identified as the cause of infections in ICUs. All ST859-SCC*mec* IV/t969 strains analyzed in the current study were *agr* I positive and invariably resistant to penicillin, ampicillin, and ciprofloxacin. There is evidence that ST859-SCC*mec* IV had a short history in Iran. A study conducted by Japoni-Nejad et al. in Iran to determine the molecular characterization of CA-MRSA isolates, 154 isolates of *S*. *aureus* from the anterior nares of 700 healthy students. Data obtained from this study showed sequence types ST22, ST25, ST859, ST14, and ST15. In their study, the ST859-SCC*mec* IV/t325 clone was *she* positive and resistant to tetracycline and fusidic acid [3]. Although the ST859-SCC*mec* IV clone has previously been reported as a CA-MRSA clone, it has recently been reported as PVL-negative HA-MRSA in Iran. The presence of this specific strain in both the community and hospitals suggests that CA-MRSA clones have successfully established themselves in hospital settings and are actively circulating.

The ST291-SCC*mec* IV/t030 clone was the third most-common clone among the MRSA clinical isolates. All strains related to this clone were multi-resistant and *pvl* negative. It is known that MRSA isolates belonging to this clone have been found in Asian and European countries such as England, Italy, Switzerland, Korea, India, and Lebanon [41]. The current study demonstrates that ST291 has successfully established in the ICUs of different hospitals in Tehran, although this clone was most frequently reported as isolates from the community. Similar results were reported by Havaei et al. [38] in Iran in which ST291 was detected in MRSA and MSSA strains isolated from patients with high frequency.

ST239-MRSA-III, the oldest pandemic MRSA strain, is known as the Hungarian, Vienna, Portuguese, Czech and Brazilian clone. This strain has been reported as a predominant HA-MRSA clone circulating in Iranian hospitals [3]. Harris at al. [42] reported that MRSA ST239/*spa* t037 is an ancestral ST239 *spa* type and may descend from ST8 and ST30 parents via the acquisition of SCC*mec* type III and recombination of a large portion of the sequence, including the arcC locus. The current results indicated that multi-resistant ST239-SCC*mec* III/t037 clones were the major circulating clone in hospital A in Tehran. They were also wide-spread among patients hospitalized in the ICU. Shahsavan et al. [29] reported that MRSA clone ST239 occurred at a prevalence of 82% in clinical isolates and all strains were resistant to ciprofloxacin, erythromycin, gentamicin, tetracycline, and trimethoprim-sulfamethoxazole.

Previous studies have divided ST239-MRSA-III into three clades; South American, European, and Asian clades. There is also evidence that the multi-resistant ST239 clone is predominant in the USA, Europe, and some Asian countries [10, 42]. It appears that the ST239-SCC*mec* III/t037 clone circulating in Iranian hospitals could have transferred from neighboring countries. It has been reported that ST6 has a relatively high prevalence in Asian countries such as Oman, the UAE, and Korea [32], but the prevalence of ST6 was low in the current study. Collectively, these findings show that all ST6 strains were PVL negative; however, further studies are required in this regard.

The present study revealed a high rate of non-invasive CA-MRSA (74.3%) that was distributed among 11 different clones. Of these clones, the three epidemic clones ST22-SCC*mec* IV/t790 (15.7%), ST859-SCC*mec* IV/t969 (14.3%), and ST239-SCC*mec* III/t037 (15.7%), predominated among non-invasive MRSA isolates. The current study also indicated that nine molecular types caused invasive infections, the most common of which were ST22-SCC*mec* IV/t790 (7.1%), ST859-SCC*mec* IV/t969 (4.3%), and ST6-SCC*mec* IV/t034 (4.3%). It has been established that regional differences exist in the distribution of invasive and non-invasive MRSA isolates.

A study conducted in China to investigate clinical and molecular characteristics of invasive CA *S*. *aureus* infections in Chinese children detected a total of 25 STs. Invasive MRSA was mainly comprised of ST59 (69%), whereas the most frequent molecular types for invasive MSSA were ST88 (16.3%), ST25 (14.1%), ST7 (14.1%), ST2155 (13%), and ST188 (9.8%) [43]. Ilczyszyn et al. reported that two epidemic clones (CC5-MRSA-II or CC45-MRSA-IV) were the most common molecular types identified among invasive MRSA isolates. They also showed that the majority (43%) of isolates belonged to *spa* types t003, t084, and t091 [44, 45]. In the present study, isolates in *agr* group II were prevalent in invasive infections and *agr* group I in noninvasive infections. This was largely in accordance with the findings reported by Rasmussen et al., who reported isolates within *agr* II were significantly associated with invasive disease.

Although the majority of studies showed an association between invasive diseases of different molecular types, it is reasonable to assume that certain *S*. *aureus* clonal lineages harboring specific sets of virulence and resistance genes are more successful at causing an invasive disease. Nevertheless, it is noteworthy that some studies have failed to show an association between invasive disease and clonality [43–45].

## Conclusion

To summarize, our study revealed that the clinical MRSA isolates belonged to diverse genetic backgrounds nevertheless four distinct genotypes of MRSA strains were identified among hospitalized patients in ICU. According to the MLST, *spa* typing, *agr* and SCC*mec* typing, ST22-SCC*mec* IV/t790 clone with a high level of antimicrobial resistance is a prototype clone in Iranian hospitals. Continuous and nationwide MRSA surveillance studies are necessary to investigate clonal distribution of MRSA in community to hospitals.
